# The CRISPR/Cas9 System Delivered by Extracellular Vesicles

**DOI:** 10.3390/pharmaceutics15030984

**Published:** 2023-03-18

**Authors:** Xinglong Zhu, Mengyu Gao, Yongfeng Yang, Weimin Li, Ji Bao, Yi Li

**Affiliations:** 1 Key Laboratory of Transplant Engineering and Immunology, Institute of Clinical Pathology, West China Hospital, Sichuan University, Chengdu 610041, China; 2 Institute of Respiratory Health, West China Hospital, Sichuan University, Chengdu 610041, China; 3 Precision Medicine Key Laboratory, West China Hospital, Sichuan University, Chengdu 610041, China; 4 Department of Respiratory and Critical Care Medicine, West China Hospital, Sichuan University, Chengdu 610041, China

**Keywords:** CRISPR/Cas9, extracellular vesicles, delivery, biomedical applications

## Abstract

Clustered regularly interspaced short palindromic repeat (CRISPR)/CRISPR-associated protein (Cas) systems can precisely manipulate DNA sequences to change the characteristics of cells and organs, which has potential in the mechanistic research on genes and the treatment of diseases. However, clinical applications are restricted by the lack of safe, targeted and effective delivery vectors. Extracellular vesicles (EVs) are an attractive delivery platform for CRISPR/Cas9. Compared with viral and other vectors, EVs present several advantages, including safety, protection, capacity, penetrating ability, targeting ability and potential for modification. Consequently, EVs are profitably used to deliver the CRISPR/Cas9 in vivo. In this review, the advantages and disadvantages of the delivery form and vectors of the CRISPR/Cas9 are concluded. The favorable traits of EVs as vectors, such as the innate characteristics, physiological and pathological functions, safety and targeting ability of EVs, are summarized. Furthermore, in terms of the delivery of the CRISPR/Cas9 by EVs, EV sources and isolation strategies, the delivery form and loading methods of the CRISPR/Cas9 and applications have been concluded and discussed. Finally, this review provides future directions of EVs as vectors of the CRISPR/Cas9 system in clinical applications, such as the safety, capacity, consistent quality, yield and targeting ability of EVs.

## 1. Introduction

In the 1970s, with the development of genetic engineering, a breakthrough dawned upon genome editing technology, which was a powerful tool for implementing fixed-point editing DNA sequences [1]. To date, genome editing tools have provided a new horizon for biomedical research, such as gene biology, biological mechanisms and treatment of disease [2]. The application of genome editing tools can be traced back to 1971, when specific DNA fragments were successfully manipulated by restriction enzymes [3]. With advances in research on endonucleases integrated with locators, a number of genome editing tools have been developed, for example, zinc finger nucleases, transcription activator-like effector nucleases and clustered regularly interspaced short palindromic repeat (CRISPR)/CRISPR-associated protein (Cas) systems [4].

For genome editing, the discovery of CRISPR/Cas systems has ushered revolutionized breakthroughs. In the study of defensive mechanisms of bacteria and archaea, CRISPR/Cas could be depicted as a defensive mechanism similar to the adaptive immune system against foreign invading nucleic acids, which was initially discovered [4,5]. When foreign invading nucleic acids enter, the acquired nucleic acid fragments are integrated and processed and finally generate CRISPR RNA (crRNA), which can complementarily pair with foreign invading nucleic acid sequences and guide the Cas protein, then form a complex with the Cas protein and other RNA for shearing DNA [6]. Because the type II CRISPR/Cas9 system is simpler and a single Cas9 protein can cleave nucleic acid segments, the CRISPR/Cas9 system is widely used in genome editing among the numerous discovered CRISPR/Cas systems. In practical applications, a single artificial synthetic guide RNA (sgRNA), formed by the combination of crRNA and trans-activating crRNA (tracrRNA), has the ability of targeted binding and Cas9 shears DNA. The CRISPR/Cas9 system has been commonly used in basic biology, biomedicine and agriculture research [2,3,7].

To implement genome editing via CRISPR/Cas9, it is necessary to consider how to deliver it [8]. Generally, the strategies of delivering CRISPR/Cas9 are classified into two major classes (viral and nonviral vector) [9]. The delivery efficiency of CRISPR/Cas9 and gene editing efficiency can be significantly increased by viral vectors [10]. Regrettably, the intrinsic factors of viral vectors, including safety, high cost, difficult operation and limited packaging capacity, restrict their application [11,12]. Nonviral vectors are mainly based on physical methods and synthetic chemical and natural materials, such as microinjection, electroporation, liposomes, nanoparticles, and gold nanoparticles [13,14]. Although these vectors possess simple operations, cost-effectiveness and universality, their cytotoxicity, limited penetrability and imprecise targeting delivery limit their further development [8,14]. Consequently, in the process of CRISPR/Cas9 delivery, the ways in which to overcome problems of safety, efficiency, stability, targeting, penetrability and biocompatibility should be focused on. Meanwhile, novel gene delivery systems should be developed.

Extracellular vesicles (EVs), secreted by a variety of cells, possess a phospholipid bilayer-enclosed structure, which was initially described as a “cellular junk” [15,16]. Although EVs can be roughly classified into exosomes, microvesicles and apoptotic bodies by size and generation methods, it is difficult to isolate pure EVs due to their overlapping size, biomarkers and composition [16,17]. Therefore, exosomes, microvesicles and apoptotic bodies are uniformly called EVs in this review. With advances in EV research, EVs have been shown to act as indispensable messengers in intercellular communication [18]. Meanwhile, since EVs are involved in various physiological and pathological processes by regulating the immune response, tissue repair and cell growth, the potential value of EVs has been revealed in the field of diagnostic biomarkers and therapy [10,19]. EVs can effectively transport lipids, proteins and nucleic acids (for instance, mRNA, miRNA, lncRNA, circular RNA, ribosomal RNA, tRNA, and DNA fragments) [20]. In addition, recent studies have shown that EVs can be used as vectors to deliver biomolecules, especially in gene delivery systems [12].

**Figure 1:**
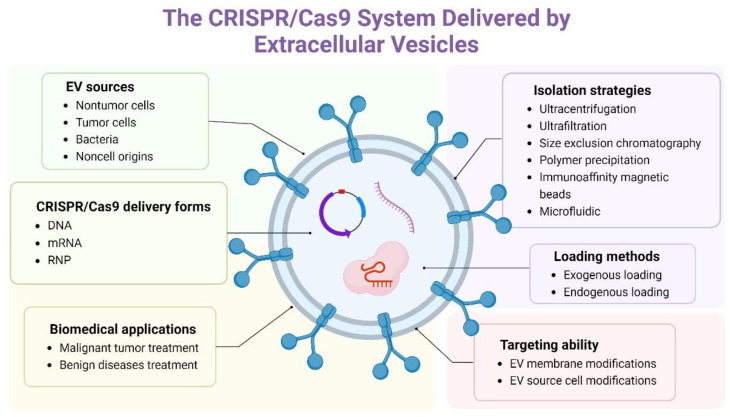
Key factors of extracellular vesicles (EVs) delivering the CRISPR/Cas9 system.

In this review, the delivery form and vectors of the CRISPR/Cas9 system are concluded and compared; the advantages of EVs as vectors are mainly summarized; the delivery of the CRISPR/Cas9 system by EVs, including EV sources and isolation strategies, the delivery form and loading methods of the CRISPR/Cas9 system and applications in vitro and in vivo have been concluded and discussed. The CRISPR/Cas9 system delivered by EVs has potential in clinical disease treatments (Figure 1).

## 2. Delivery of CRISPR/Cas9

Because the molecular weight of the Cas9 protein is 160 kDa and the gRNA is negatively charged, these characteristics of the gRNA and Cas9 protein make it difficult for them to cross the cell membrane [12,21]. In the CRISPR/Cas9 gene editing system in vitro, the sgRNA and Cas9 protein should be delivered into cells to avoid degradation and move into the nucleus for gene editing [22]. Furthermore, in the more complex microenvironment in vivo, the CRISPR/Cas9 system may be degraded and neutralized by physical, chemical and biological obstacles and the immune system [10,23]. Therefore, to improve the efficiency, safety and targeting of gene editing, the delivery form of the CRISPR/Cas9 system and how to deliver the CRISPR/Cas9 system should be seriously considered.

In general, the delivery form of the CRISPR/Cas9 system can be classified as DNA, RNA or ribonucleoprotein complexes (RNPs) [12,24]. The RNP is defined as the sgRNA compounded with Cas9 protein in vitro [25]. Through plasmids carrying DNA sequences of gRNA and Cas9 protein, the CRISPR/Cas9 system is delivered into cells. This method is economical as well as convenient [12,26]. However, gRNA and Cas9 protein are further formed by transcription and translation in cells, which increases the working time and the rate of off-target effects [27]. Meanwhile, this process, which is difficult to control, could have potential risks for DNA sequences integrated into the genome [28]. Since transcription is not needed, the delivery of the RNA form of the CRISPR/Cas9 system can effectively decrease the editing efficiency [29]. Unfortunately, RNA is easily degraded, which decreases the efficiency of gene editing [30]. Gene editing immediately begins to work through the delivery of the RNP form of the CRISPR/Cas9 system in the nucleus, which leads to a higher efficiency of gene editing and a lower rate of off-target effects than DNA and RNA forms of the CRISPR/Cas9 system [31]. RNPs only temporarily exist in cells, which is beneficial for curing genetic and infectious disorders. Nevertheless, the delivery of RNPs is more costly than that of DNA and RNA [12,32].

In vitro, the efficiency of gene editing and the rate of off-target effects are mainly discussed, which is simpler than in vivo. For gene editing via CRISPR/Cas9 in vivo, several key factors should fit the following criteria: (1) safety (delivery products and strategies); (2) crossing the physiological and pathological barriers (dense characteristics of some tissue, acid and enzymes microenvironment, blood-embryo and blood–brain barrier); (3) targeting (effectively targeted tissue or organ); and (4) accurate gene editing (improving the gene editing efficiency and avoiding damage to the genome) [10,33].

For delivery of the CRISPR/Cas9 system, these strategies can be classified into viral and nonviral vectors [34]. Adeno-associated viruses (AAVs), Adenoviruses (AVs), Lentiviruses (LVs) and Baculoviruses which are some typical viral vectors, have been exploited for delivering gene editing systems [8,9]. These viral vectors can efficiently deliver the CRISPR/Cas9 system into cells crossing physiological barriers and the cell membrane and possess high transfection and gene editing efficiency [35]. However, especially in vivo, several inherent characteristics of viruses, for example, immunogenicity inducing immune responses and viral genomes randomly inserting into host genomes, have caused significant safety problems [8]. Compared with other viral vectors, AAV is safer since it cannot replicate, does not integrate into genomes and has low immunogenicity in vivo [36,37]. Nonetheless, the delivery of the CRISPR/Cas9 system may be compromised by the limited packaging capacity of AAV [38].

Electroporation and microinjection, as classical nonviral physical methods, have been used to deliver the CRISPR/Cas9 system in vitro [35]. Electroporation depends on currents, which can create numerous transient small pores in cell membranes; then, the CRISPR/Cas9 system can be delivered into cells [9]. This method is inexpensive, easy to perform and is high throughput, but the conditions of electroporation are highly variable, and cells are vulnerable [8]. Microinjection relies on microscopes and needles to inject RNPs, which are used in zygotes for the generation of small animal models. Because of the high cost of microinjection, delicate operation, low throughput and inappropriateness of in vivo editing, the applications of microinjection have been limited [39,40].

Lipid-based synthetic chemical materials have been frequently used for the delivery of the CRISPR/Cas9 system [14]. In particular, a variety of commercial liposomes have been developed. For example, liposomes can effectively deliver the DNA, RNA and RNP form of the CRISPR/Cas9 system [10,41]. With a hydrophobic tail and hydrophilic head, liposomes can spontaneously form the structure of lipid bilayers [42]. The CRISPR/Cas9 system can be encapsulated by liposomes or combined with liposomes to form complexes, and then liposome–CRISPR/Cas9 complexes fuse with cell membranes to enter cells [35]. Unlike viral vectors, liposomes are a safer method since their delivery mechanism is based on endocytosis [10]. Meanwhile, compared with electroporation, liposomes are milder to cells. Therefore, liposomes have harmonious characteristics in all aspects, including inherent structure, biocompatibility, low immunogenicity and low toxicity [9]. Gold nanoparticles can deliver the CRISPR/Cas9 system in vivo and in vitro. The advantage of gold nanoparticles is that they are biologically inert, so the immune response is hardly triggered [43,44].

In conclusion, all delivery forms of the CRISPR/Cas9 system and delivery strategies have their own benefits and drawbacks (Table 1). According to the purpose and means of research, the appropriate forms and strategies are selected.

## 3. Several Key Factors of EVs as Vectors

EVs can be generated and secreted by eukaryotes and prokaryotes and then transfer biomacromolecules (such as nucleic acids, proteins, lipids, glycoproteins, etc.) for exchange of information [18,20]. In vivo, EVs are associated with several physiological and pathophysiological processes (for instance, the immune system, viral pathogenicity, etc.) and various diseases (including liver, cardiovascular, tumor, lung and central nervous system) [16,19,45]. Consequently, EVs can reveal the biological status of biological fluids, which provides new insight for diagnosing diseases. Meanwhile, the ability of EVs to carry information indicates that EVs can deliver cargoes for therapeutics [46]. Given the loading capacity of various biomacromolecules, ability to target specific cells, immunoregulation and ease of engineering, EVs can be an ideal vector for the delivery of therapeutic agents [47]. Furthermore, these beneficial characteristics of EVs are gradually being used in CRISPR/Cas9 delivery, which has potential for gene editing in vivo [48].

### 3.1. Innate Characteristics of EVs

EVs are generated by several cell types. According to the EV biogenesis mechanism, EVs can be classified into exosomes, microvesicles and apoptotic bodies [16]. In general, exosomes are released by the plasma membrane fused with multivesicular bodies (MVBs), which are formed by an inward budding of the endosome, and microvesicles and apoptotic bodies are formed by an outward budding of the plasma membrane under different conditions [49]. Consequently, the EV membrane features of a phospholipid bilayer-enclosed structure containing various transmembrane and membrane-anchored proteins depend on the EV biogenesis mechanism [15,17]. These features are beneficial to the targeting and uptake of EVs.

For cargo delivery, EVs have advantages with respect to protection and capacity. With large molecules, hydrophilicity and different charges, naked nucleic acids and proteins have difficulty crossing cell membranes [10]. They were consequently encapsulated by EVs for delivery and protection. Furthermore, the EV membrane structure can effectively protect naked nucleic acids and proteins from serum endonucleases and the immune system [48]. Compared with other vectors, EVs are unique in that they can deliver different types of cargoes (saccharides, nucleic acids, proteins and lipids) [18]. A number of functional biomacromolecules, especially nucleic acids and proteins, are exogenously encapsulated and carried by EVs. In the process of EV biogenesis, several biomacromolecules related to cell communication, disease pathogenesis and therapeutic agents are innately encapsulated in EVs [16,17].

Several natural biological barriers, such as dense tissue (tumor and cartilage), acidic and enzymatic microenvironments (intestinal mucosa and gastric) and vascular endothelial cell barriers (blood-embryo barrier and blood-brain barrier (BBB)), can obstruct the delivery of cargoes in vivo [10,50]. With high biocompatibility and natural generation, EVs can cross these natural biological barriers efficiently to deliver cargoes [16,51]. The tumor microenvironment, especially high-density tumor tissue, seriously affects tumor penetration, which limits the application of nanomedicine [50]. Liposomes can permeate into the tumor tissue to deliver cargoes. However, the permeability of liposomes is mainly affected by the size, charge, type of cargo and type of tumor, which limits the applications of liposomes [52]. EVs derived from tumor cells can effectively permeate into the tumor. Sánchez et al. showed that the expression of metalloproteinases was increased, the extracellular matrix was degraded and EV penetration was improved when EV surface proteins (CD147, tetraspanin Tspan8 or CD44) and miRNAs (miRNA-494, miRNA-542-3p, and miRNA-21-5p) were overexpressed [53]. Kim et al. successfully utilized tumor-derived EVs carrying the CRISPR/Cas9 system to permeate into tumor tissue for gene editing [54]. The intestinal mucosa can hinder the uptake of several vectors that can deliver cargoes [10]. The study revealed that with small size and negative charge, these vectors performed better in permeation [55]. With their small size, negative charge and stable structure, EVs derived from milk can be easily absorbed by the intestine [56,57]. Meanwhile, EVs derived from epithelial cells also permeate across the intestine [58]. A number of biomacromolecules (curcumin and various types of RNA) could be protected by EVs from digestive juices [10]. The BBB is one of the most important natural biological barriers that can selectively allow some small molecules to cross the endothelial membrane and prevent the passage of causative agents or toxins [59]. In this way, the BBB, as a protective mechanism, also obstructs the delivery of drugs and other biomacromolecules into the brain. Nevertheless, EVs can encapsulate these drugs and biomacromolecules to cross the BBB, and their penetrable efficiency depends on the size and composition of EVs [10,60]. Morad et al. successfully showed that tumor-derived EVs could cross the BBB in vivo, which was based on the endocytosis pathway. In this process, EVs decreased the expression of Rab7 in endothelial cells to promote their transport [61].

### 3.2. Physiological and Pathological Functions of EVs

With extensive distribution and generation, EVs carry various biomacromolecules to orderly shuttle in vivo, which regulates numerous physiologic processes [16]. Especially in terms of immunoregulation, EVs, generated by B lymphocytes, can present antigens by histocompatibility complex (MHC) proteins located on the EV surface [62]. Recent studies have focused on the immune-regulatory functions of EVs in tumors. According to reports, tumor cell-derived EVs exhibit the ability of immunoregulation to support cancer progression or display antitumor effects [63,64]. Hoshino et al. showed that in tumor metastasis, tumor cell-derived EVs were fused with organ-specific resident cells (fibroblasts, epithelial cells, etc.) to prepare the niche through exosomal integrins, which could lead to Src phosphorylation and proinflammatory S100 expression, facilitating tumor metastasis [65]. Nevertheless, several studies have demonstrated that tumor cell-derived EVs could carry tumor antigens and then promote tumor immunity through CD8(+) and CD4(+) T cells, which could kill tumor cells and frustrate the progression of tumors [64].

Furthermore, EVs can regulate multiple intercellular communications that are related to the cell cycle (proliferation and apoptosis), tissue repair (angiogenesis and remodeling) and regeneration [16]. Since they contain a variety of coagulants, growth factors, inflammatory factors, cytokines, RNA and lipids, platelet-derived EVs regulate the clotting response, cell cycle and tissue repair and regeneration [66]. Kim et al. demonstrated that the proliferation of human umbilical vein endothelial cells (HUVECs) could be stimulated by lipids in platelet-derived EVs [67]. Meanwhile, Zhang et al. showed that in a rat model of femoral nonunion, bone marrow mesenchymal stem cell (BM-MSC)-derived EVs could effectively promote angiogenesis and bone repair. These EVs could be taken up by HUVECs, which facilitated the proliferation of HUVECs in vitro [68].

EVs are also involved in the pathological processes related to tumors and chronic and infectious diseases [16]. In the field of tumor research, EVs, which are generated from tumor, stromal, and immune cells, carry multiple DNA, RNA, protein and other metabolites to participate in tumor development, drug resistance, metastasis and immunity [63]. Tumor cell-derived EVs can affect the fate of EV-generating tumor cells and neighboring tumor and stromal cells [63]. Raimondo et al. demonstrated that chronic myeloid leukemia-derived EVs could promote the proliferation of tumor cells through the activation of antiapoptotic pathways [69]. Nedawi et al. discovered that in glioma, only a small fraction of tumor cells expressing EGFRvIII could generate EGFRvIII-carrying EVs to neighboring tumor cells, which could activate the anchorage-independent growth of neighboring tumor cells through an activation of the AKT pathway [70]. Furthermore, Antonyak et al. showed that MDA-MB-231 and U87 glioma cell-derived EVs could confer onto normal fibroblasts and epithelial cells with several characteristics of tumor cells [71].

Recent studies have also shown that EVs are involved in other chronic and infectious diseases, such as pulmonary fibrosis (PF), cardiovascular diseases and viral infection [16,19,72]. PF, as a chronic lung disease, is characterized by an increase in fibroblasts and extracellular matrix. The pathology of PF is unknown, but EVs that are associated with it have been proven [19]. Yao et al. demonstrated that with overexpression of miR-328, M2 macrophage-derived EVs promoted the proliferation of fibroblasts in vitro and the progression of PF in vivo [73]. The types of EVs, cargoes and cell types determine the effect of EVs in the process of atherothrombosis. In general, EVs play an indispensable role in every step of atherosclerosis, including lesion initiation, plaque progression, plaque rupture and thrombosis [72]. Some related RNA virus studies have shown that infected cell-derived EVs can deliver several biomacromolecules to regulate pathological processes associated with the infection process and cellular responses [16].

### 3.3. Safety of EVs

Compared with viral vectors (AAVs, AVs and LVs) and chemical materials (liposomes and synthetic nanoparticles), EVs have several innate characteristics (such as generation from natural sources and low immunogenicity and cytotoxicity) [17]. Kamerkar et al. showed that compared with liposomes, the retention of EVs was improved by reducing phagocytosis by CD47-mediated protection in vivo. Furthermore, fibroblast-derived EVs carrying siRNA targeting oncogenic KRAS inhibited tumor development better than liposomes without an apparent immune response in vivo [74].

However, in the applications of EVs as therapeutic agents or cargo vectors in vivo, the safety of EVs must be thoroughly considered, such as the EV source, the cell culture conditions and the EV isolation strategies [12]. In terms of EV-generating cell sources, the type of cell determines the characteristics of the EV membrane and cargoes, which can affect the immune response, cell transformation and cell invasion [17]. MSCs are one of the most common EV-generating cell sources. A number of studies have proven the low immunogenicity of MSC-derived EVs in vivo [75]. Mendt et al. proved that when MSC-derived EVs were repeatedly administered in mice, the toxicity and immunoreaction of EVs were not obviously observed in vivo [76]. Furthermore, Zhu et al. also demonstrated that HEK293T cell-derived EVs could not elicit an immune response or toxicity in vivo [77]. However, since tumor cell-derived EVs contain multiple tumor-associated biomacromolecules that have the ability to potentially affect tumor development, the safety of these EVs is controversial [12]. Several studies have shown that in animal models of melanoma, liver tumors and colon carcinoma, tumor cell-derived EVs can inhibit tumor development through the T-cell-dependent immune response [63]. However, tumor cell-derived EVs can improve the proliferation of tumor cells, angiogenesis and metastasis and inhibit apoptosis to support tumor progression [12,78]. Consequently, an appropriate EV-generating cell source can guarantee the safety of EV applications. Moreover, the toxicity and hypoimmunogenicity of EVs should be further discussed in different application scenarios.

At present, EVs are mainly generated by transformed cell lines [12]. To fulfill the clinical demand of EV large-scale production and high quality, EV-generating cell culture conditions should comply with good manufacturing practice (GMP) to guarantee the consistent EV phenotype of different batches [79,80,81]. The cell culture conditions include the medium components (factors and serum), pH and cell culture type (adherent and suspension culture), which may affect the membrane structure and cargo of EVs [82]. Furthermore, EV isolation strategies also affect the quality and safety of EVs, which will be further discussed later.

### 3.4. Targeting Ability of EVs

In terms of in vivo delivery strategies of biomacromolecules and drugs, especially in CRISPR/Cas9 gene editing systems, the lack of tissue-specific vectors restricts their further clinical application [33]. EVs can not only encapsulate and protect cargoes but also achieve organ, tissue and cell targeting. In general, several molecules can lead to cell-specific homing, which connects with the targeting ability of EVs [48,83]. Consequently, the natural targeting ability of EVs is determined by the cell type. The glycans, proteins and lipids located in the EV membrane determine which organs, tissues and cells are targeted by EVs [17]. Hoshino et al. showed that EVs derived from lung, liver and brain tumor cells could be preferentially taken up by lung fibroblasts, Kupffer cells and brain endothelial cells, respectively. Furthermore, this study proved that the EV integrins α_6_β_4_ and α_6_β_1_ are associated with lung tropism, while integrin α_v_β_5_ is associated with liver tropism [65]. Meanwhile, Kim et al. demonstrated that compared with HEK293 cell-derived EVs, SKOV3 cell-derived EVs selectively accumulated in ovarian tumors in vivo, which was likely related to cell tropism [54]. Wan et al. showed that hepatic stellate cell-derived EVs could target the liver, but there were no EVs in other organs, such as the heart, spleen, lung and kidney [33].

Specific cell types confer a certain targeting ability of EVs. However, in the applications of EVs in vivo, the poor and unstable targeting ability and off-target effects are still the principal challenges [84]. Recent studies have proposed that specific membrane modification of EVs with ligands can improve their targeting ability [17]. In general, EV modification can be classified into two broad categories: (1) direct modification of the EV membrane and (2) the generation of EVs by modifying source cells. In the former strategy, for example, Zhuang et al. showed that HEK293T cell-derived EVs were directly modified with valency-controlled tetrahedral DNA nanostructures (TDNs) conjugated with DNA aptamers, which could specifically target the liver via cholesterol anchoring [84]. In the latter strategy, Xu et al. developed EVs with a chimeric antigen receptor (CAR), which was anti-CD19, derived from anti-CD19-CAR-HEK293T cells transfected with anti-CD19CAR LVs. Consequently, these EVs could target B-cell malignancies in vivo [85].

## 4. Delivery of the CRISPR/Cas9 System by EVs

With precise site-specific gene editing, the CRISPR-Cas9 system has gradually become a powerful tool to cure a number of genetic disorders and tumors, especially transthyretin amyloidosis, which has entered phase 1 clinical trials [27]. As mentioned above, EVs, with good biocompatibility, low immunogenicity, capacity, protection ability, modification and targeting ability, have emerged as favorable tissue-specific vectors for delivering the CRISPR-Cas9 system and other biomacromolecules, which have been discussed. In this section, we will focus on the delivery of the CRISPR/Cas9 system by EVs, including the EV source, the isolation strategies of EVs, the delivery form of the CRISPR/Cas9 system delivered by EVs, the loading methods of the CRISPR/Cas9 system into EVs and the experimental applications of the delivery of the CRISPR/Cas9 system by EVs (Table 2).

### 4.1. EV Sources

We previously discussed that the EV source determines the membrane and cargoes of EVs, which can affect the safety (toxicity and immunogenicity) and targeting ability of EVs [17]. Therefore, EV sources have been explored in the field of the delivery of the CRISPR/Cas9 system by EVs. In general, EV sources can be classified into four broad categories: (1) nontumor cells; (2) tumor cells; (3) bacteria; and (4) noncell origins. Among nontumor cells, with high EV yields, less abundant endogenous cargoes and simple culture, the human embryonic kidney cell lines HEK293 and HEK293T are widely used to generate EVs to deliver the CRISPR/Cas9 system [54,86]. Moreover, these cells are easily modified to produce engineered EVs. For example, after transfection with Cas9-sgRNA plasmids, HEK293 and HEK293T cells could produce EVs carrying RNPs to improve the efficiency of gene editing, and anti-CD19 CAR-HEK293T cells could generate anti-CD19 CAR EVs to improve tissue targeting ability [85,87]. Currently, numerous studies have proven the nontoxicity and low immunogenicity of HEK293 and HEK293T cell-derived EVs in vivo. However, the targeting ability of HEK293 and HEK293T cell-derived EVs is poor. Lainšček et al. demonstrated that after 3 h of intravenous injection into mice, HEK293 cell-derived EVs were rapidly distributed throughout the body, such as in the heart, lung, spleen, kidney and brain, and especially in the liver. With blood circulation, the EVs were still mainly distributed in the liver but were not distributed in the heart 24 h after injection [87]. Moreover, EVs can be generated by hepatic stellate cells and red blood cells to deliver the CRISPR/Cas9 system. Hepatic stellate cell-derived EVs were safe and could target the liver [33]. Usman et al. showed that with the lack of nuclear and mitochondrial DNA, the most easily available, abundant amount and safety of clinical applications, red blood cells could deliver CRISPR/Cas9 to realize gene editing [88]. MSC-derived EVs can also deliver CRISPR/Cas9 for gene editing [91].

Tumor cells, such as SKOV3, A549 and B16-F10 cells, are also used to generate EVs to deliver the CRISPR/Cas9 system [54,95]. Because of the source of tumor cells, the toxicity and immunogenicity of EVs are of serious concern. Kim et al. showed that, compared with lipopolysaccharide or CpG oligodeoxynucleotides, SKOV3 cell-derived EVs only led to human peripheral blood mononuclear cells generating a minor production of TNF-α and INF-α. Meanwhile, due to cell tropism, these EVs could target SKOV3 xenografts in vivo [54]. Furthermore, Ye et al. demonstrated that in vivo gene editing, A549 and B16-F10 cell-derived EVs could mainly accumulate in the liver. In particular, B16-F10 cell-derived EVs carried ITGβ5, which is associated with liver tropism [95].

Bacteria, such as Halobacterium, can also produce EVs that have been used to deliver the CRISPR/Cas9 system [92]. Liu et al. presented the idea that through bacteria as factories, EVs carrying RNPs and specific ligands could be produced, which is low-cost and efficient [97]. There are serum-derived EVs (noncell origin) that can deliver the CRISPR/Cas9 system. Serum-derived EVs lack immunogenicity and are easily obtained. Majeau et al. demonstrated that intravenous and intramuscular injection of serum-derived EVs did not cause adverse effects, such as inflammatory reactions, within 7 days in mice [96].

### 4.2. Isolation of EVs

The quality, safety, purity and yield of EVs can be controlled by isolation strategies, which is important for clinical applications in vivo. With their small size, low density and mixture with analogs (lipoprotein complexes, cell fragments and protein aggregates), the isolation of EVs is enormously challenging [80,81]. Moreover, due to the overlap of size and similar morphological characteristics, the segregation of EV subtypes, including exosomes, microvesicles and apoptotic bodies, becomes more difficult [98]. The purity, yield and integrity of EVs can be affected by the different isolation strategies [16]. Consequently, standardized and appropriate isolation strategies should be further developed. Generally, according to the principle of separation, isolation strategies can be classified into four broad categories: (1) the density of EVs (ultracentrifugation); (2) the size of EVs (ultrafiltration and size exclusion chromatography); (3) the solubility of EVs (polymer precipitation); and (4) the immunoaffinity of EVs (immunoaffinity magnetic beads).

Ultracentrifugation (UC), as the gold standard EV isolation method, is the most common strategy, accounting for almost half of EV isolation [99]. According to the density and size of EVs and their analogs, UC can remove cells, fragments and contaminants. UC possesses several advantages, for example, easy operation, low cost and largescale [98]. However, UC takes considerable time, is low throughput, cannot remove nucleic acids, lipoproteins and proteins and leads to the highly variable purity of EVs [16]. Based on the molecular size, ultrafiltration (UF) utilizes size-exclusion membranes to separate higher purity EVs than UC. However, in the process of EV isolation, the filtration membrane may be blocked by the samples, and the shear forces can destroy the membrane integrity of EVs [81]. The principle of size exclusion chromatography (SEC) is similar to that of UF [16]. Because of the porous beads, the elution speed of smaller particles is slower than that of larger particles, which can effectively protect the integrity of EVs with high purity but can lead to EVs mixed with contaminants of similar size [100]. The polymer precipitation method is used in the isolation of less soluble substances, such as EVs [98]. At present, the reagents used in the polymer precipitation method include protamine, acetate and polyethylene (PEG). With inexpensive, gentle and simple characteristics, PEG has been widely used to separate EVs, which leads to the low purity of EVs [81]. According to the immunoaffinity of EVs, immunoaffinity magnetic beads can isolate high-purity EVs. However, this method is expensive and has low yields [100]. In addition to the above methods, microfluidic approaches have also been used to isolate EVs [101]. With the development of EVs, numerous kits for EV isolation have become commercially available [81].

EV application in terms of delivering the CRISPR/Cas9 system. At present, EVs are almost completely isolated by UC. In addition, Lin et al. utilized a 500 × 10^−3^ M NaCl solution containing 12% PEG 6000 to separate EVs [89]. Majeau and coauthors isolated serum EVs by a size exclusion column (SEC qEV10/70 nm) and further purified them by UF [96].

### 4.3. The Delivery Form of the CRISPR/Cas9 System and the Loading Methods

The delivery form of the CRISPR/Cas9 system determines the efficiency of gene editing. Briefly, because they avoid the process of transcription and translation, RNPs make gene editing faster and more efficient and lower the rate of off-target effects than the DNA and RNA forms of the CRISPR/Cas9 system, but are more expensive [27]. Recent studies have demonstrated that the DNA, RNA and RNP form of the CRISPR/Cas9 system can be carried and delivered by EVs for gene editing in vitro and in vivo. For example, tumor cell-derived EVs could carry CRISPR/Cas9 plasmids to inhibit poly (ADP-ribose) polymerase-1 (PARP-1) [54]. Moreover, to improve the capacity of EVs, Lin et al. developed hybrid nanoparticles made up of EVs and liposomes [89]. Usman et al. showed that red blood cell-derived EVs could carry CRISPR/Cas9 mRNA for genome editing, and the efficiency of mRNA was higher than that of plasmids [88]. Moreover, HEK293T cell-derived EVs could also carry CRISPR/Cas9 RNPs for gene editing [86].

On the other hand, the delivery form of the CRISPR/Cas9 system can also affect the loading methods of the CRISPR/Cas9 system. Successful encapsulation of the CRISPR/Cas9 system by EVs plays a vital role in gene editing [93]. In contrast to other vectors, EVs should be intact after loading of the CRISPR/Cas9 system. The loading methods of the CRISPR/Cas9 system can be classified into two broad categories: (1) exogenous loading (directly loading the CRISPR/Cas9 system by electroporation, incubation, transfection and sonication on EVs) and (2) endogenous loading (through transforming cells to produce EVs with the loading CRISPR/Cas9 system). At present, CRISPR/Cas9 plasmids and mRNA can only be loaded by exogenous loading methods (such as electroporation and transfection kits) into EVs [54,88,89]. CRISPR/Cas9 RNPs can be loaded by exogenous loading methods (electroporation, sonication and transfection) and endogenous loading methods into EVs [33,84,86,96]. In terms of endogenous cargoes, loading cargoes of large molecular weights (such as CRISPR/Cas9 RNPs) remains challenging and under exploration [94]. Therefore, several studies have been devoted to enriching RNPs into EVs through specific interactions between modified RNPs and EVs. Wang et al. developed EVs containing arrestin domain containing protein 1 (ARRDC1), which could interact with WW-domain-containing proteins. Consequently, Cas9 was linked with WW domains to improve the enrichment of Cas9 in EVs, which did not affect the activity and functions of the Cas9 protein [86]. Yao et al. demonstrated that by utilizing the interaction between RNA aptamers and aptamer-binding proteins (ABPs), RNPs could be enriched in EVs [90]. Moreover, myristoylated Cas9 was beneficial for accumulation in EVs, which led to RNP enrichment in the EVs [93]. The RNPs could also be enriched by light-induced protein heterodimerization techniques in the EVs [94]. In addition to the above loading methods, the CRISPR/Cas9 system can potentially be loaded into EVs by the anthrax lethal toxin. Anthrax lethal toxin consists of protective antigen (PA) and lethal factor (LF). PA can form a channel to recruit and transfer LF. Based on this transporter system, foreign proteins fused with the N-terminus of LF can be delivered into cells by this system, including the Cas9 protein [102]. Meanwhile, LF is delivered not only into the cytosol but also into extracellular vesicles (EVs), which can load various cargoes into EVs, such as LFn-DTA, siRNA, ASOs and Cas9 protein [103,104].

### 4.4. The Application of Delivery of the CRISPR/Cas9 System by EVs

With a number of EV advantages, including safety, protection, capacity, penetrating ability, targeting ability and potential for modification, EVs are more preferably used to deliver the CRISPR/Cas9 system in vivo than in vitro [105]. At present, EVs carrying the CRISPR/Cas9 system have been used in tumor and other disease therapy.

#### 4.4.1. Malignant Tumor Treatment

Traditional tumor treatment strategies, such as surgery, chemotherapy and radiotherapy, only effectively cure some patients and can lead to serious side effects and poor prognosis. CRISPR/Cas9 technology brings hope to tumor therapy [5,106]. Due to its connection with the DNA damage response, PARP-1 has become a promising target of tumor therapy, especially for breast and ovarian tumors [107]. Kim et al. showed that through intravenous injection of CRISPR/Cas9-loaded EVs into SKOV3 xenograft mice, PARP-1 could be effectively inhibited, reducing the tumor volume and weight [54]. Moreover, the inhibition of PARP-1 improved chemosensitivity to cisplatin. The oncogenic mutant Kras^G12D^ can improve the proliferation of pancreatic tumors by Ras signaling [108]. McAndrews et al. demonstrated that MSC-derived EVs could carry and deliver the CRISPR/Cas9 plasmid to knock out the mutant Kras^G12D^ oncogenic allele in KPC689 cells (derived from an autochthonous pancreatic tumor of mice), which could effectively suppress the growth of tumors in subcutaneous and orthotopic KPC689 graft mice [91]. With high overexpression in liver tumor tissue and cells, the WNT10B gene could be inhibited by siRNA to inhibit the viability and migration of HepG2 cells [109]. Zhuang et al. developed EVs modified with TDNs that could accumulate in HepG2 cells and liver tumor organoids in vitro. Meanwhile, CRISPR/Cas9 RNPs were delivered by EVs to decrease the expression of WNT10B in HepG2 xenograft mice, resulting in the inhibition of tumor growth [84]. K (lysine) acetyltransferase 5 (KAT5) plays an indispensable role in the growth of hepatocellular carcinoma. Consequently, the inhibition of KAT5 can suppress tumor growth [110]. Wan et al. showed that LX-2 cell-derived EVs could encapsulate and deliver CRISPR/Cas9 RNPs to suppress KAT5 expression in an orthotopic Huh-7 xenograft murine model, hence reducing KAT5 expression, inhibiting tumor growth and prolonging mouse survival [33]. In almost 30% of human tumors, such as Burkitt’s lymphoma and other hematological malignancies, overexpression of the MYC oncogene has been detected [85]. Xu et al. developed EVs modified with anti-CD19 CAR that could accumulate in tumor tissue and deliver the CRISPR/Cas9 plasmid into subcutaneous Raji xenograft mice, leading to MYC mutation, which could induce apoptosis. Therefore, the tumor volume decreased compared with that of the control [85].

#### 4.4.2. Benign Disease Treatment

CRISPR/Cas9 technology has gradually become a potential treatment strategy for genetic diseases and other diseases by inactivating or correcting disease-causing genes [3]. Hepatocyte growth factor (HGF) plays a crucial role in liver regeneration. Lainšček and coauthors showed that the *Hgf* gene could be improved by CRISPR/Cas9 to target the first exon in vitro. Furthermore, the alpha-naphthylisothiocyanate-induced liver damage murine model was administered EVs carrying CRISPR/Cas9 plasmids, leading to an increased HGF to induce liver regeneration and decreased ALT, bile acids, bilirubin and cholesterol [87]. The p53 upregulated modulator of apoptosis (PUMA) is associated with acetaminophen (APAP)-induced liver injury [111]. Wan and coauthors showed that, through EVs carrying CRISPR/Cas9 RNPs to inhibit the expression of PUMA protein in an APAP-induced liver injury murine model, the PUMA protein, AST and ALT levels, the number of apoptotic and necrotic cells and hyperemia could be markedly reduced, and the survival of mice was prolonged [33]. Cyclin E1 (CcnE1) can enhance the proliferation of hepatic stellate cells and is connected with liver fibrogenesis. Through the siRNA inhibition of CcnE1, the occurrence and development of fibrosis can be inhibited [112]. The CCl_4_-induced chronic liver fibrosis murine model was treated with EVs carrying CRISPR/Cas9 RNPs targeting CcnE1, leading to the expression of CcnE1 and α-SMA protein being reduced. Furthermore, the initiation and development of liver fibrosis were attenuated [33]. Duchenne muscular dystrophy (DMD), caused by various mutations in the *Dmd* gene, is one of the most common genetic diseases and can reduce the expression of the dystrophin protein [113]. Majeau et al. demonstrated that with the nonsense mutation of *Dmd* gene-exon 23, *mdx* mice were injected with EVs carrying CRISPR/Cas9 RNPs to delete exons 23 and 24. Compared with the control, exons 23 and 24 were effectively deleted, and the expression of dystrophin protein was restored in muscles. Moreover, in *hDMD/mdx* mice expressing the human *Dmd* gene, the *Dmd* genes could also be modified [96].

## 5. Conclusions and Future Directions

The gene editing system (CRISPR/Cas9) can precisely manipulate DNA sequences to change the characteristics of cells and organs, which has potential in mechanistic research on genes and the treatment of diseases [4]. For example, in human clinical trials, genetic diseases, such as transthyretin amyloidosis (NCT04601051) and Leber congenital amaurosis (NCT03872479), are treated by CRISPR/Cas9 in vivo editing. The delivery vectors are proprietary lipid nanoparticles (LNPs) and AAVs [3,114]. Especially in terms of the treatment of clinical diseases, the CRISPR/Cas9 system should be accurately and safely delivered to the cells and organs that are needed for gene editing. Consequently, the need for appropriate vectors is urgent for clinical application [3]. At present, EVs carrying therapeutic cargoes are being evaluated for disease treatment in human clinical trials. For example, MSC-derived EVs transfected with miR-124 have been used to treat ischemic stroke (NCT03384433), and MSC-derived EVs loaded with miRNA against mutant KRAS have been used to treat pancreatic cancer (NCT03608631) [115]. However, there are no clinical trials on CRISPR/Cas9 delivered by EVs. Compared with viral and other vectors, with good biocompatibility, capacity and physiological properties, EVs are gradually regarded as the preferable “truck” with GPS carrying the CRISPR/Cas9 system for targeted delivery [48]. In this review, the delivery of the CRISPR/Cas9 system (such as vectors and the delivery form of the CRISPR/Cas9 system), the traits of EVs as vectors (including innate characteristics, physiological and pathological functions, safety and targeting ability) and the delivery of the CRISPR/Cas9 system by EVs (such as EV source and isolation strategies, the delivery form and loading methods of the CRISPR/Cas9 system and applications) have been concluded and discussed.

Ideally, with a high efficiency of gene editing and without safety problems, EVs can encapsulate CRISPR/Cas9 RNPs to target cells or tissue. At present, several studies have shown that EVs can carry and deliver the CRISPR/Cas9 system to edit genes. However, greater efforts to overcome some problems, for instance, the safety, capacity, consistent quality, yield and targeting ability of EVs, are needed. First, because of the biogenesis of EVs, a number of EV biomacromolecules have the potential to affect the physiological functions of cells. In particular, tumor-derived EVs show the ability to target tumors but contain several tumor-related molecules that have the potential to lead to tumor development and metastasis [54]. Therefore, the safety and source of EVs should be further investigated and developed. Second, the capacity of EVs and loading efficiency of CRISPR/Cas9 RNPs, which affect the efficiency of delivery and gene editing, have been mentioned. Hybrid EVs fused with other materials and enhancing the specific interaction between modified RNPs and EVs are full of prospects for improving the capacity of EVs [86]. Third, the consistent quality and yield of EVs play an indispensable role during the process of EV clinical applications, which determines the concordance and stability of EV batch to batch and the abundant supply of EVs. The stability of the EV source and isolation and further purification strategies determine the quality and yield of EVs [81]. Therefore, improving the stability of EV sources and building standard isolation and further purification strategies should be seriously considered. Fourth, the targeting ability of EVs is critical to the precise gene editing of CRISPR/Cas9 in vivo. However, the precise targeting ability of EVs, which is determined by cell tropism, is limited. At present, the targeting ability of EVs is being enhanced. There is a bright future in modifying cells to produce modified EVs with ligands and directly modifying EVs with ligands [17]. Furthermore, the advent of new CRISPR-based systems (base editors, primer editors and RNA-targeting Cas13) can remarkably improve the safety, precision and efficacy of gene editing [1]. Therefore, in the near future, with the development of EVs, genome editing can be used in clinical disease treatments.

## Figures and Tables

**Table 1:** Comparing different delivery strategies of the CRISPR/Cas9 system, these strategies can be classified into viral and nonviral vectors. AAVs: adeno-associated viruses; AVs: adenoviruses; LVs: lentiviruses; LSCMs: lipid-based synthetic chemical materials; EVs: extracellular vesicles (EVs); RNP: ribonucleoprotein complex. + denotes low; ++ denotes medium; +++ denotes high.

Delivery Vectors	Viral Vectors			Nonviral Vectors				
	AAVs	AVs	LVs	Electroporation	Microinjection	LSCMs	Gold Nanoparticles	EVs
Safety	+++	++	+	+++	+++	++	++	+++
Capacity	+	++	+	−	−	++	++	+++
The form of the CRISPR/Cas9 system	DNA	DNA	DNA	DNA, RNA, RNP	DNA, RNA, RNP	DNA, RNA, RNP	RNP	DNA, RNA, RNP
Targeting ability	++	−	−	−	−	−	−	+++
Permeation	+++	+++	+++	−	−	++	++	+++
Delivery efficiency	+++	+++	+++	+++	+	++	++	+++
Applications	In vivo	In vitro and in vivo	In vitro and in vivo	In vitro	In vitro	In vitro and in vivo	In vitro and in vivo	In vitro and in vivo
Cost	+++	+++	++	+	+++	++	++	+++

**Table 2:** The CRISPR-Cas9 system delivered by EVs. UC: ultracentrifugation; SEC: size exclusion chromatography.

EV Source	Isolation Strategies	Delivery Form of CRISPR/Cas9 System	Loading Methods of CRISPR/Cas9 System	Targeting Ability	Applications	References
SKOV3 cells and HEK293 cells	UC	Plasmids	Electroporation	The tumor of SKOV3 xenograft mice	Treatment of ovarian tumor	[54]
HEK293T cells	UC	RNPs	Endogenous loading	None targeting ability	Editing cells in vitro	[86]
HEK293 cells	UC	RNPs	Endogenous loading	None targeting ability	Treatment of liver damage	[87]
Red blood cells	UC	mRNA	Electroporation	None targeting ability	Editing cells in vitro	[88]
HEK293FT cells	Polymer precipitation	Plasmids	Transfection kit	None targeting ability	Editing cells in vitro	[89]
HEK293T cells	UC	RNPs	Endogenous loading	None targeting ability	Editing cells in vitro and in vivo	[25]
HEK293T cells	UC	RNPs	Endogenous loading	None targeting ability	Editing cells in vitro	[90]
HEK293T cells	UC	RNPs	Sonication	The tumor of HepG2 xenograft mice	Treatment of liver tumor	[84]
HEK293T cells and MSCs	UC	Plasmids	Transfection kit	None targeting ability	Treatment of pancreatic tumor	[91]
Halobacterium	Centrifugation	Plasmids	Incubation	None targeting ability	Editing cells in vitro	[92]
HEK293T cells	UC	RNPs	Endogenous loading	None targeting ability	Editing cells in vitro	[93]
Expi293F cells	UC	RNPs	Endogenous loading	None targeting ability	Editing cells in vitro	[94]
LX-2 cells	UC	RNPs	Electroporation	The liver tissue	Treatment of acute liver injury, chronic liver fibrosis and hepatocellular carcinoma	[33]
A549 cells and B16-F10 cells	UC	RNPs	Endogenous loading	None targeting ability	Editing cells in vitro and in vivo	[95]
Serum	SEC	RNPs	Transfection kit	None targeting ability	Treatment of Duchenne muscular dystrophy	[96]
Anti-CD19-CAR-HEK293T cells	UC	Plasmids	Electroporation	The tumor of Raji xenograft mice	Treatment of B-cell malignancies	[85]

## Data Availability

Not applicable.

## References

[1] Doudna J.A. (2020). The promise and challenge of therapeutic genome editing. Nature.

[2] Yang L., Chen J. (2020). A Tale of Two Moieties: Rapidly Evolving CRISPR/Cas-Based Genome Editing. Trends Biochem. Sci..

[3] Pickar-Oliver A., Gersbach C.A. (2019). The next generation of CRISPR-Cas technologies and applications. Nat. Rev. Mol. Cell Biol..

[4] Hille F., Richter H., Wong S.P., Bratovic M., Ressel S., Charpentier E. (2018). The Biology of CRISPR-Cas: Backward and Forward. Cell.

[5] Wang S.W., Gao C., Zheng Y.M., Yi L., Lu J.C., Huang X.Y., Cai J.B., Zhang P.F., Cui Y.H., Ke A.W. (2022). Current applications and future perspective of CRISPR/Cas9 gene editing in cancer. Mol. Cancer.

[6] Knott G.J., Doudna J.A. (2018). CRISPR-Cas guides the future of genetic engineering. Science.

[7] Baghini S.S., Gardanova Z.R., Abadi S.A.H., Zaman B.A., Ilhan A., Shomali N., Adili A., Moghaddar R., Yaseri A.F. (2022). CRISPR/Cas9 application in cancer therapy: A pioneering genome editing tool. Cell. Mol. Biol. Lett..

[8] Yin H., Kauffman K.J., Anderson D.G. (2017). Delivery technologies for genome editing. Nat. Rev. Drug. Discov..

[9] Yip B.H. (2020). Recent Advances in CRISPR/Cas9 Delivery Strategies. Biomolecules.

[10] Jiang X.C., Zhang T., Gao J.Q. (2022). The in vivo fate and targeting engineering of crossover vesicle-based gene delivery system. Adv. Drug. Deliv. Rev..

[11] Song X., Liu C., Wang N., Huang H., He S., Gong C., Wei Y. (2021). Delivery of CRISPR/Cas systems for cancer gene therapy and immunotherapy. Adv. Drug. Deliv. Rev..

[12] Kostyushev D., Kostyusheva A., Brezgin S., Smirnov V., Volchkova E., Lukashev A., Chulanov V. (2020). Gene Editing by Extracellular Vesicles. Int. J. Mol. Sci..

[13] Taha E.A., Lee J., Hotta A. (2022). Delivery of CRISPR-Cas tools for in vivo genome editing therapy: Trends and challenges. J. Control. Release.

[14] Li L., Hu S., Chen X. (2018). Non-viral delivery systems for CRISPR/Cas9-based genome editing: Challenges and opportunities. Biomaterials.

[15] Kalluri R., LeBleu V.S. (2020). The biology, function, and biomedical applications of exosomes. Science.

[16] Liu S., Wu X., Chandra S., Lyon C., Ning B., Jiang L., Fan J., Hu T.Y. (2022). Extracellular vesicles: Emerging tools as therapeutic agent carriers. Acta Pharm. Sin. B.

[17] Meng W., He C., Hao Y., Wang L., Li L., Zhu G. (2020). Prospects and challenges of extracellular vesicle-based drug delivery system: Considering cell source. Drug. Deliv..

[18] Zhao Y., Li X., Zhang W., Yu L., Wang Y., Deng Z., Liu M., Mo S., Wang R., Zhao J. (2021). Trends in the biological functions and medical applications of extracellular vesicles and analogues. Acta Pharm. Sin. B.

[19] Yang Y., Huang H., Li Y. (2022). Roles of exosomes and exosome-derived miRNAs in pulmonary fibrosis. Front. Pharmacol..

[20] Massaro C., Sgueglia G., Frattolillo V., Baglio S.R., Altucci L., Dell’Aversana C. (2020). Extracellular Vesicle-Based Nucleic Acid Delivery: Current Advances and Future Perspectives in Cancer Therapeutic Strategies. Pharmaceutics.

[21] Jinek M., Jiang F., Taylor D.W., Sternberg S.H., Kaya E., Ma E., Anders C., Hauer M., Zhou K., Lin S. (2014). Structures of Cas9 endonucleases reveal RNA-mediated conformational activation. Science.

[22] Li G., Liu Y.G., Chen Y. (2019). Genome-editing technologies: The gap between application and policy. Sci. China Life Sci..

[23] Liu S., Cheng Q., Wei T., Yu X., Johnson L.T., Farbiak L., Siegwart D.J. (2021). Membrane-destabilizing ionizable phospholipids for organ-selective mRNA delivery and CRISPR-Cas gene editing. Nat. Mater..

[24] Lino C.A., Harper J.C., Carney J.P., Timlin J.A. (2018). Delivering CRISPR: A review of the challenges and approaches. Drug. Deliv..

[25] Montagna C., Petris G., Casini A., Maule G., Franceschini G.M., Zanella I., Conti L., Arnoldi F., Burrone O.R., Zentilin L. (2018). VSV-G-Enveloped Vesicles for Traceless Delivery of CRISPR-Cas9. Mol. Ther. Nucleic Acids.

[26] Kosicki M., Tomberg K., Bradley A. (2018). Repair of double-strand breaks induced by CRISPR-Cas9 leads to large deletions and complex rearrangements. Nat. Biotechnol..

[27] Huang K., Zapata D., Tang Y., Teng Y., Li Y. (2022). In vivo delivery of CRISPR-Cas9 genome editing components for therapeutic applications. Biomaterials.

[28] Kim S., Kim D., Cho S.W., Kim J., Kim J.S. (2014). Highly efficient RNA-guided genome editing in human cells via delivery of purified Cas9 ribonucleoproteins. Genome Res..

[29] Kowalski P.S., Rudra A., Miao L., Anderson D.G. (2019). Delivering the Messenger: Advances in Technologies for Therapeutic mRNA Delivery. Mol. Ther..

[30] Mitschka S., Mayr C. (2022). Context-specific regulation and function of mRNA alternative polyadenylation. Nat. Rev. Mol. Cell Biol..

[31] Zhang S., Shen J., Li D., Cheng Y. (2021). Strategies in the delivery of Cas9 ribonucleoprotein for CRISPR/Cas9 genome editing. Theranostics.

[32] Vakulskas C.A., Dever D.P., Rettig G.R., Turk R., Jacobi A.M., Collingwood M.A., Bode N.M., McNeill M.S., Yan S., Camarena J. (2018). A high-fidelity Cas9 mutant delivered as a ribonucleoprotein complex enables efficient gene editing in human hematopoietic stem and progenitor cells. Nat. Med..

[33] Wan T., Zhong J., Pan Q., Zhou T., Ping Y., Liu X. (2022). Exosome-mediated delivery of Cas9 ribonucleoprotein complexes for tissue-specific gene therapy of liver diseases. Sci. Adv..

[34] Xu X., Liu C., Wang Y., Koivisto O., Zhou J., Shu Y., Zhang H. (2021). Nanotechnology-based delivery of CRISPR/Cas9 for cancer treatment. Adv. Drug. Deliv. Rev..

[35] Liu C., Zhang L., Liu H., Cheng K. (2017). Delivery strategies of the CRISPR-Cas9 gene-editing system for therapeutic applications. J. Control. Release.

[36] Wang D., Zhang F., Gao G. (2020). CRISPR-Based Therapeutic Genome Editing: Strategies and In Vivo Delivery by AAV Vectors. Cell.

[37] Verdera H.C., Kuranda K., Mingozzi F. (2020). AAV Vector Immunogenicity in Humans: A Long Journey to Successful Gene Transfer. Mol. Ther..

[38] McClements M.E., MacLaren R.E. (2017). Adeno-associated Virus (AAV) Dual Vector Strategies for Gene Therapy Encoding Large Transgenes. Yale J. Biol. Med..

[39] Horii T., Arai Y., Yamazaki M., Morita S., Kimura M., Itoh M., Abe Y., Hatada I. (2014). Validation of microinjection methods for generating knockout mice by CRISPR/Cas-mediated genome engineering. Sci. Rep..

[40] Long C., McAnally J.R., Shelton J.M., Mireault A.A., Bassel-Duby R., Olson E.N. (2014). Prevention of muscular dystrophy in mice by CRISPR/Cas9-mediated editing of germline DNA. Science.

[41] Alghuthaymi M.A., Ahmad A., Khan Z., Khan S.H., Ahmed F.K., Faiz S., Nepovimova E., Kuca K., Abd-Elsalam K.A. (2021). Exosome/Liposome-like Nanoparticles: New Carriers for CRISPR Genome Editing in Plants. Int. J. Mol. Sci..

[42] Pensado A., Seijo B., Sanchez A. (2014). Current strategies for DNA therapy based on lipid nanocarriers. Expert Opin. Drug Deliv..

[43] Lee K., Conboy M., Park H.M., Jiang F., Kim H.J., Dewitt M.A., Mackley V.A., Chang K., Rao A., Skinner C. (2017). Nanoparticle delivery of Cas9 ribonucleoprotein and donor DNA in vivo induces homology-directed DNA repair. Nat. Biomed. Eng..

[44] Glass Z., Li Y., Xu Q. (2017). Nanoparticles for CRISPR-Cas9 delivery. Nat. Biomed. Eng..

[45] Garikipati V.N.S., Shoja-Taheri F., Davis M.E., Kishore R. (2018). Extracellular Vesicles and the Application of System Biology and Computational Modeling in Cardiac Repair. Circ. Res..

[46] Le Q.V., Lee J., Lee H., Shim G., Oh Y.K. (2021). Cell membrane-derived vesicles for delivery of therapeutic agents. Acta Pharm. Sin. B..

[47] Du R., Wang C., Zhu L., Yang Y. (2022). Extracellular Vesicles as Delivery Vehicles for Therapeutic Nucleic Acids in Cancer Gene Therapy: Progress and Challenges. Pharmaceutics.

[48] Liang Y., Iqbal Z., Wang J., Xu L., Xu X., Ouyang K., Zhang H., Lu J., Duan L., Xia J. (2022). Cell-derived extracellular vesicles for CRISPR/Cas9 delivery: Engineering strategies for cargo packaging and loading. Biomater. Sci..

[49] He C., Zheng S., Luo Y., Wang B. (2018). Exosome Theranostics: Biology and Translational Medicine. Theranostics.

[50] Ding J., Chen J., Gao L., Jiang Z., Zhang Y., Li M., Xiao Q., Lee S.S., Chen X. (2019). Engineered nanomedicines with enhanced tumor penetration. Nano Today.

[51] Holder B., Jones T., Shimizu V.S., Rice T.F., Donaldson B., Bouqueau M., Forbes K., Kampmann B. (2016). Macrophage Exosomes Induce Placental Inflammatory Cytokines: A Novel Mode of Maternal–Placental Messaging. Traffic.

[52] Yamamoto S., Kato A., Sakurai Y., Hada T., Harashima H. (2017). Modality of tumor endothelial VEGFR2 silencing-mediated improvement in intratumoral distribution of lipid nanoparticles. J. Control. Release.

[53] Sánchez C.A., Andahur E.I., Valenzuela R., Castellón E.A., Fullá J.A., Ramos C.G., Triviño J.C. (2016). Exosomes from bulk and stem cells from human prostate cancer have a differential microRNA content that contributes cooperatively over local and pre-metastatic niche. Oncotarget.

[54] Kim S.M., Yang Y., Oh S.J., Hong Y., Seo M., Jang M. (2017). Cancer-derived exosomes as a delivery platform of CRISPR/Cas9 confer cancer cell tropism-dependent targeting. J. Control. Release.

[55] Lamson N.G., Berger A., Fein K.C., Whitehead K.A. (2020). Anionic nanoparticles enable the oral delivery of proteins by enhancing intestinal permeability. Nat. Biomed. Eng..

[56] Warren M.R., Zhang C., Vedadghavami A., Bokvist K., Dhal P.K., Bajpayee A.G. (2021). Milk exosomes with enhanced mucus penetrability for oral delivery of siRNA. Biomater. Sci..

[57] Liao Y., Du X., Li J., Lonnerdal B. (2017). Human milk exosomes and their microRNAs survive digestion in vitro and are taken up by human intestinal cells. Mol. Nutr. Food Res..

[58] Carobolante G., Mantaj J., Ferrari E., Vllasaliu D. (2020). Cow Milk and Intestinal Epithelial Cell-derived Extracellular Vesicles as Systems for Enhancing Oral Drug Delivery. Pharmaceutics.

[59] Sweeney M.D., Zhao Z., Montagne A., Nelson A.R., Zlokovic B.V. (2019). Blood-Brain Barrier: From Physiology to Disease and Back. Physiol. Rev..

[60] Ashizawa A.T., Holt J., Faust K., Liu W., Tiwari A., Zhang N., Ashizawa T. (2019). Intravenously Administered Novel Liposomes, DCL64, Deliver Oligonucleotides to Cerebellar Purkinje Cells. Cerebellum.

[61] Morad G., Carman C.V., Hagedorn E.J., Perlin J.R., Zon L.I., Mustafaoglu N., Park T.E., Ingber D.E., Daisy C.C., Moses M.A. (2019). Tumor-Derived Extracellular Vesicles Breach the Intact Blood-Brain Barrier via Transcytosis. ACS Nano.

[62] Liu J., Jiang F., Jiang Y., Wang Y., Li Z., Shi X., Zhu Y., Wang H., Zhang Z. (2020). Roles of Exosomes in Ocular Diseases. Int. J. Nanomed..

[63] Zhang L., Yu D. (2019). Exosomes in cancer development, metastasis, and immunity. Biochim. Biophys. Acta Rev. Cancer.

[64] Kurywchak P., Tavormina J., Kalluri R. (2018). The emerging roles of exosomes in the modulation of immune responses in cancer. Genome Med..

[65] Hoshino A., Costa-Silva B., Shen T.L., Rodrigues G., Hashimoto A., Mark M.T., Molina H., Kohsaka S., Di Giannatale A., Ceder S. (2015). Tumour exosome integrins determine organotropic metastasis. Nature.

[66] Johnson J., Wu Y.W., Blyth C., Lichtfuss G., Goubran H., Burnouf T. (2021). Prospective Therapeutic Applications of Platelet Extracellular Vesicles. Trends Biotechnol..

[67] Kim H.K., Song K.S., Chung J.H., Lee K.R., Lee S.N. (2004). Platelet microparticles induce angiogenesis in vitro. Br. J. Haematol..

[68] Zhang L., Jiao G., Ren S., Zhang X., Li C., Wu W., Wang H., Liu H., Zhou H., Chen Y. (2020). Exosomes from bone marrow mesenchymal stem cells enhance fracture healing through the promotion of osteogenesis and angiogenesis in a rat model of nonunion. Stem Cell Res. Ther..

[69] Raimondo S., Saieva L., Corrado C., Fontana S., Flugy A., Rizzo A., De Leo G., Alessandro R. (2015). Chronic myeloid leukemia-derived exosomes promote tumor growth through an autocrine mechanism. Cell Commun. Signal..

[70] Al-Nedawi K., Meehan B., Micallef J., Lhotak V., May L., Guha A., Rak J. (2008). Intercellular transfer of the oncogenic receptor EGFRvIII by microvesicles derived from tumour cells. Nat. Cell Biol..

[71] Antonyak M.A., Li B., Boroughs L.K., Johnson J.L., Druso J.E., Bryant K.L., Holowka D.A., Cerione R.A. (2011). Cancer cell-derived microvesicles induce transformation by transferring tissue transglutaminase and fibronectin to recipient cells. Proc. Natl. Acad. Sci. USA.

[72] Giro O., Jimenez A., Pane A., Badimon L., Ortega E., Chiva-Blanch G. (2021). Extracellular vesicles in atherothrombosis and cardiovascular disease: Friends and foes. Atherosclerosis.

[73] Yao M.Y., Zhang W.H., Ma W.T., Liu Q.H., Xing L.H., Zhao G.F. (2019). microRNA-328 in exosomes derived from M2 macrophages exerts a promotive effect on the progression of pulmonary fibrosis via FAM13A in a rat model. Exp. Mol. Med..

[74] Kamerkar S., LeBleu V.S., Sugimoto H., Yang S., Ruivo C.F., Melo S.A., Lee J.J., Kalluri R. (2017). Exosomes facilitate therapeutic targeting of oncogenic KRAS in pancreatic cancer. Nature.

[75] Wu P., Zhang B., Shi H., Qian H., Xu W. (2018). MSC-exosome: A novel cell-free therapy for cutaneous regeneration. Cytotherapy.

[76] Mendt M., Kamerkar S., Sugimoto H., McAndrews K.M., Wu C.C., Gagea M., Yang S., Blanko E.V.R., Peng Q., Ma X. (2018). Generation and testing of clinical-grade exosomes for pancreatic cancer. JCI Insight.

[77] Zhu X., Badawi M., Pomeroy S., Sutaria D.S., Xie Z., Baek A., Jiang J., Elgamal O.A., Mo X., Perle K. (2017). Comprehensive toxicity and immunogenicity studies reveal minimal effects in mice following sustained dosing of extracellular vesicles derived from HEK293T cells. J. Extracell. Vesicles.

[78] Rahman M.A., Barger J.F., Lovat F., Gao M., Otterson G.A., Nana-Sinkam P. (2016). Lung cancer exosomes as drivers of epithelial mesenchymal transition. Oncotarget.

[79] Elahi F.M., Farwell D.G., Nolta J.A., Anderson J.D. (2020). Preclinical translation of exosomes derived from mesenchymal stem/stromal cells. Stem Cells.

[80] Feng Z.Y., Zhang Q.Y., Tan J., Xie H.Q. (2022). Techniques for increasing the yield of stem cell-derived exosomes: What factors may be involved?. Sci. China Life Sci..

[81] Chen J., Li P., Zhang T., Xu Z., Huang X., Wang R., Du L. (2021). Review on Strategies and Technologies for Exosome Isolation and Purification. Front. Bioeng. Biotechnol..

[82] Whitford W., Guterstam P. (2019). Exosome manufacturing status. Future Med. Chem..

[83] Rana S., Yue S., Stadel D., Zoller M. (2012). Toward tailored exosomes: The exosomal tetraspanin web contributes to target cell selection. Int. J. Biochem. Cell Biol..

[84] Zhuang J., Tan J., Wu C., Zhang J., Liu T., Fan C., Li J., Zhang Y. (2020). Extracellular vesicles engineered with valency-controlled DNA nanostructures deliver CRISPR/Cas9 system for gene therapy. Nucleic Acids Res..

[85] Xu Q., Zhang Z., Zhao L., Qin Y., Cai H., Geng Z., Zhu X., Zhang W., Zhang Y., Tan J. (2020). Tropism-facilitated delivery of CRISPR/Cas9 system with chimeric antigen receptor-extracellular vesicles against B-cell malignancies. J. Control. Release.

[86] Wang Q., Yu J., Kadungure T., Beyene J., Zhang H., Lu Q. (2018). ARMMs as a versatile platform for intracellular delivery of macromolecules. Nat. Commun..

[87] Lainscek D., Kadunc L., Keber M.M., Bratkovic I.H., Romih R., Jerala R. (2018). Delivery of an Artificial Transcription Regulator dCas9-VPR by Extracellular Vesicles for Therapeutic Gene Activation. ACS Synth. Biol..

[88] Usman W.M., Pham T.C., Kwok Y.Y., Vu L.T., Ma V., Peng B., Chan Y.S., Wei L., Chin S.M., Azad A. (2018). Efficient RNA drug delivery using red blood cell extracellular vesicles. Nat. Commun..

[89] Lin Y., Wu J., Gu W., Huang Y., Tong Z., Huang L., Tan J. (2018). Exosome-Liposome Hybrid Nanoparticles Deliver CRISPR/Cas9 System in MSCs. Adv. Sci..

[90] Yao X., Lyu P., Yoo K., Yadav M.K., Singh R., Atala A., Lu B. (2021). Engineered extracellular vesicles as versatile ribonucleoprotein delivery vehicles for efficient and safe CRISPR genome editing. J. Extracell. Vesicles.

[91] McAndrews K.M., Xiao F., Chronopoulos A., LeBleu V.S., Kugeratski F.G., Kalluri R. (2021). Exosome-mediated delivery of CRISPR/Cas9 for targeting of oncogenic Kras(G12D) in pancreatic cancer. Life Sci. Alliance.

[92] Gao R., Luo Q., Li Y., Song L., Cai J.S., Xiong Y., Yan F., Liu J. (2022). Biosynthetic Nanobubble-Mediated CRISPR/Cas9 Gene Editing of Cdh2 Inhibits Breast Cancer Metastasis. Pharmaceutics.

[93] Whitley J.A., Kim S., Lou L., Ye C., Alsaidan O.A., Sulejmani E., Cai J., Desrochers E.G., Beharry Z., Rickman C.B. (2022). Encapsulating Cas9 into extracellular vesicles by protein myristoylation. J. Extracell. Vesicles.

[94] Osteikoetxea X., Silva A., Lazaro-Ibanez E., Salmond N., Shatnyeva O., Stein J., Schick J., Wren S., Lindgren J., Firth M. (2022). Engineered Cas9 extracellular vesicles as a novel gene editing tool. J. Extracell. Vesicles.

[95] Ye Y., Shi Q., Yang T., Xie F., Zhang X., Xu B., Fang J., Chen J., Zhang Y., Li J. (2022). In Vivo Visualized Tracking of Tumor-Derived Extracellular Vesicles Using CRISPR-Cas9 System. Technol. Cancer Res. Treat..

[96] Majeau N., Fortin-Archambault A., Gerard C., Rousseau J., Yameogo P., Tremblay J.P. (2022). Serum extracellular vesicles for delivery of CRISPR-CAS9 ribonucleoproteins to modify the dystrophin gene. Mol. Ther..

[97] Liu Y., Smid E.J., Abee T., Notebaart R.A. (2019). Delivery of genome editing tools by bacterial extracellular vesicles. Microb. Biotechnol..

[98] Li P., Kaslan M., Lee S.H., Yao J., Gao Z. (2017). Progress in Exosome Isolation Techniques. Theranostics.

[99] Gandham S., Su X., Wood J., Nocera A.L., Alli S.C., Milane L., Zimmerman A., Amiji M., Ivanov A.R. (2020). Technologies and Standardization in Research on Extracellular Vesicles. Trends Biotechnol..

[100] Zhang Y., Bi J., Huang J., Tang Y., Du S., Li P. (2020). Exosome: A Review of Its Classification, Isolation Techniques, Storage, Diagnostic and Targeted Therapy Applications. Int. J. Nanomed..

[101] Le M.N., Fan Z.H. (2021). Exosome isolation using nanostructures and microfluidic devices. Biomed. Mater..

[102] Hirschenberger M., Stadler N., Fellermann M., Sparrer K.M.J., Kirchhoff F., Barth H., Papatheodorou P. (2021). CRISPA: A Non-viral, Transient Cas9 Delivery System Based on Reengineered Anthrax Toxin. Front. Pharmacol..

[103] Benedita F., Simon R. (2022). Engineering protein toxins to modulate the intracellular trafficking of biologics into exosomes for third order drug targeting. Eur. J. Extracell. Vesicles.

[104] Simon R., Benedita F. (2020). Method for Preparing Liposomes. International Patent.

[105] Duan L., Ouyang K., Wang J., Xu L., Xu X., Wen C., Xie Y., Liang Y., Xia J. (2021). Exosomes as Targeted Delivery Platform of CRISPR/Cas9 for Therapeutic Genome Editing. ChemBioChem.

[106] Katti A., Diaz B.J., Caragine C.M., Sanjana N.E., Dow L.E. (2022). CRISPR in cancer biology and therapy. Nat. Rev. Cancer.

[107] Michels J., Vitale I., Saparbaev M., Castedo M., Kroemer G. (2014). Predictive biomarkers for cancer therapy with PARP inhibitors. Oncogene.

[108] Bryant K.L., Mancias J.D., Kimmelman A.C., Der C.J. (2014). KRAS: Feeding pancreatic cancer proliferation. Trends Biochem. Sci..

[109] Wu G., Fan X., Sun L. (2015). Silencing of Wnt10B reduces viability of heptocellular carcinoma HepG2 cells. Am. J. Cancer Res..

[110] Kwan S.Y., Sheel A., Song C.Q., Zhang X.O., Jiang T., Dang H., Cao Y., Ozata D.M., Mou H., Yin H. (2020). Depletion of TRRAP Induces p53-Independent Senescence in Liver Cancer by Down-Regulating Mitotic Genes. Hepatology.

[111] Chen D., Ni H.M., Wang L., Ma X., Yu J., Ding W.X., Zhang L. (2019). p53 Up-regulated Modulator of Apoptosis Induction Mediates Acetaminophen-Induced Necrosis and Liver Injury in Mice. Hepatology.

[112] Bangen J.M., Hammerich L., Sonntag R., Baues M., Haas U., Lambertz D., Longerich T., Lammers T., Tacke F., Trautwein C. (2017). Targeting CCl(4) -induced liver fibrosis by RNA interference-mediated inhibition of cyclin E1 in mice. Hepatology.

[113] Happi Mbakam C., Lamothe G., Tremblay G., Tremblay J.P. (2022). CRISPR-Cas9 Gene Therapy for Duchenne Muscular Dystrophy. Neurotherapeutics.

[114] Gillmore J.D., Gane E., Taubel J., Kao J., Fontana M., Maitland M.L., Seitzer J., O’Connell D., Walsh K.R., Wood K. (2021). CRISPR-Cas9 In Vivo Gene Editing for Transthyretin Amyloidosis. N. Engl. J. Med..

[115] O’Brien K., Breyne K., Ughetto S., Laurent L.C., Breakefield X.O. (2020). RNA delivery by extracellular vesicles in mammalian cells and its applications. Nat. Rev. Mol. Cell. Biol..

